# Advancing and implementing cardiovascular drug therapy

**DOI:** 10.1093/ehjcvp/pvag086

**Published:** 2026-11-04

**Authors:** Bianca Rocca

**Affiliations:** Department of Medicine and Surgery, LUM University, S.S. 100 Km. 18 – 70010 Casamassima, Bari, Italy

Heart failure (HF) pharmacotherapy, cardiometabolic pharmacology, drug safety, environmental prevention, and future strategies in cardiovascular pharmacology are represented in this issue of the journal.

The issue opens with a clinically relevant, unresolved question despite the central role of mineralocorticoid receptor antagonists (MRAs) in guideline-directed HF therapy^1^: are eplerenone and spironolactone clinically interchangeable? In a large Swedish registry–based cohort with long-term follow-up and laboratory data, Wärnberg *et al*.^2^ report that, in contrast to spironolactone, initiation of eplerenone was associated with lower all-cause mortality, more favourable kidney outcomes, and less hyperkalaemia. These findings extend previous studies by incorporating accurate assessment of renal function and laboratory measurements. These observations also complement recent data from a Danish registry–based target-trial emulation in HF with reduced ejection fraction,^3^ reporting lower mortality among initiators of eplerenone, although treatment effects were attenuated in per-protocol analyses, suggesting the relevance of drug persistence and tolerability. Notably, these studies provide consistent findings across independent healthcare systems, patient cohorts, and analytical approaches. The ESC 2026 HF guidelines^1^ consider the MRA drug class as foundational therapy, explicitly acknowledging pharmacokinetic and pharmacodynamic differences between individual MRAs and identifying randomized comparisons between single MRAs as a gap in evidence. Wärnberg *et al*. and Svanström *et al*. expand the evidence base in this area of uncertainty. While observational data cannot definitively determine whether differences reflect drug pharmacological properties, tolerability, persistence, cardiorenal effects, or residual confounding, the consistency of the signal across independent studies provides a compelling rationale for prospective comparative trials called for by the guidelines.

Heart failure with reduced ejection fraction is associated with an increased incidence of new-onset atrial fibrillation (AF), worsening haemodynamics and major cardiovascular outcomes. Current HF guidelines recommend angiotensin receptor–neprilysin inhibitor (ARNI) based on documented benefits on HF-related outcomes.^1^ The potential impact of ARNI on incident AF remains uncertain. Park *et al*.^4^ explore this association in ∼18 000 propensity score-matched patients who either continued renin–angiotensin inhibition or switched to ARNI. Switching to ARNI vs. continuing RAS inhibitors was associated with incidence rates of 10.73 and 12.15 per 100 person-years of new-onset AF, respectively. Volpe *et al*.^5^ provide a thoughtful commentary on these findings, in the context of previous randomized and observational evidence, pathogenetic factors and the need for future studies.

Two contributions in this issue highlight the possible relevance of epigenetic pathways and environmental factors on cardiovascular therapy, in addition to drugs’ pharmacodynamics. Atzemian *et al*.^6^ review the rapidly evolving field of epigenetics and pharmaco-epigenetics, highlighting how epigenetic pathways may influence not only disease expression but also response to ‘old’ drugs. They also identify epigenetic pathways as potential targets for future ‘epi-drugs’. In a different context, Bikdeli *et al*.^7^ present the rationale and design of the Coated Or Chewable Aspirin in Patients with Established AThErosclerotic Disease and a Hybrid Strategy to Mitigate the Adverse Effects of AIR Pollution (COATED-AIR) trial. This study examines whether aspirin formulation and active mitigation of environmental air pollution in relation to drug intake may improve cardiovascular prevention by low-dose aspirin, an antiplatelet drug with a well-established benefit–risk profile. Although those contributions^6,7^ address distinct areas of cardiovascular medicine, both focus on how biological and environmental modifiers may influence the clinical therapeutic effects beyond pharmacodynamics. These concepts align with recent discussions on the contextual determinants of treatment effects in cardiovascular pharmacotherapy.^8^

Sustained and successful pharmacotherapy in daily practice requires that treatments are properly explained to and understood by patients, appropriately implemented and revised over time by healthcare professionals,^9^ and used safely, especially in populations with comorbidities and complex therapeutic regimens.^10^ Goutaudier and Hulot discuss how artificial intelligence (AI) may enhance treatment understanding through *ad hoc*, upfront AI-assisted communication of expected benefits and risks, shifting the focus from reminder-based interventions towards early strategies that improve treatment acceptance.^11^ They highlight that drug adherence is often lowered not by unwillingness to take treatment but by insufficient understanding of its value in each individual. This challenge extends beyond prescribing decisions into the way of communicating efficacy and safety evidence.

Improving safety is central for successful implementation of effective therapies. Al-Ansary *et al*.^12^ address an incompletely explored aspect of the safety of glucagon-like peptide-1 receptor agonists (GLP-1RAs), a class of drugs that has transformed the management of obesity and diabetes.^13^ As GLP-1RAs become increasingly used, robust, real-world safety data become increasingly relevant for their long-term and appropriate use. The authors investigate a mechanistically plausible but incompletely studied safety concern: whether gastrointestinal and autonomic effects of GLP-1RAs may increase vagal activity-related (VAR) adverse events, including syncope, bradyarrhythmia, cardiac device implantation, and, consequently, fractures.^12^ Using Danish health registers, they studied more than 50 000 individuals initiating GLP-1RAs, more than 73 000 initiators of sodium-glucose cotransporter-2 inhibitors (SGLT2is; as active comparator) all with type 2 diabetes (T2DM) as well as over 95 000 individuals with obesity initiating GLP-1RA alongside sex- and age-matched individuals. One-year, standardized absolute VAR risk was comparable between individuals with T2DM on GLP-1RA and SGLT2is, and it was also comparable between GLP-1RA-treated individuals with obesity and a sex- and age-matched background population. These findings provide reassurance regarding VAR adverse events and highlight the importance of continued safety evaluation of these increasingly used agents.

Moustafa *et al*.^14^ extensively review cardiac safety of commonly used antiemetic drug classes, an important but often underappreciated source of iatrogenic arrhythmias across oncology, perioperative medicine, emergency care, and general practice. Integrating mechanistic evidence, randomized studies, pharmacovigilance data, and guideline recommendations, the authors show that the risk of QT prolongation and torsades de pointes varies substantially not only between but also within antiemetic classes. Notably, the review highlights important differences among commonly used antiemetics and proposes a three-tier classification of antiemetics according to their torsadogenic risk and a practical, stepwise prescribing algorithm for patients with congenital or acquired long QT syndrome and other conditions at increased arrhythmogenic risk. This framework provides clinicians with an actionable approach, balancing effective symptom control against preventable proarrhythmic risk.

The issue concludes with a PharmaPlain summary of the SOUL trial,^15^ explaining the results of a major cardiovascular outcomes trial of oral semaglutide in plain language accessible to a broader audience. Although different in format, this contribution shares the common objective of supporting implementation of evidence-based therapies in real-world practice by improving communication between researchers, clinicians, and patients.

## Data Availability

No new data are generated by this editorial.
